# Prognostic Significance of Admission Glucose Combined with Hemoglobin A1c in Acute Ischemic Stroke Patients with Reperfusion Therapy

**DOI:** 10.3390/brainsci12020294

**Published:** 2022-02-21

**Authors:** Anmo Wang, Ting Cui, Changyi Wang, Qiange Zhu, Xuening Zhang, Shucheng Li, Yuan Yang, Wenzuo Shang, Bo Wu

**Affiliations:** 1 Center of Cerebrovascular Diseases, Department of Neurology, West China Hospital, Sichuan University, Chengdu 610041, China; 2015181622040@stu.scu.edu.cn (A.W.); cuiting@stu.scu.edu.cn (T.C.); linty.zhang@stu.scu.edu.cn (X.Z.); t21198865@163.com (S.L.); yy015055@swmu.edu.cn (Y.Y.); shangwenzuo@stu.scu.edu.cn (W.S.); 2 Department of Rehabilitation Medicine Center, West China Hospital, Sichuan University, Chengdu 610041, China; changyi11@scu.edu.cn; 3 The Second Department of Neurology, Shanxi Provincial People’s Hospital, Xi’an 710068, China; 2017224025300@stu.scu.edu.cn

**Keywords:** admission glucose, HbA1c, acute ischemic stroke, reperfusion therapy, outcome

## Abstract

Background: Elevated admission glucose and hemoglobin A1c (HbA1c) levels have been suggested to be associated with 90-day functional outcomes in acute ischemic stroke (AIS) patients with endovascular thrombectomy (EVT). However, whether the prognostic significance of admission glucose and that of HbA1c have a joint effect on patients with intravascular thrombolysis (IVT) and/or EVT remains unclear. This study aimed to explore the association between admission glucose combined with HbA1c and outcomes in patients with reperfusion therapy. Methods: Consecutive AIS patients treated with IVT and/or EVT between 2 January 2018 and 27 February 2021 in West China hospital were enrolled. Admission glucose and HbA1c levels were measured at admission. Participants were divided into four groups according to admission glucose level (categorical variable: <7.8 and ≥7.8 mmol/L) and HbA1c level (categorical variable: <6.5% and ≥6.5%): normal glucose and normal HbA1c (NGNA), normal glucose and high HbA1c (NGHA), high glucose and normal HbA1c (HGNA), and high glucose and high HbA1c (HGHA). The primary outcome was an unfavorable functional outcome defined as a modified Rankin Scale (mRS) ≥ 3. The secondary outcome was all-cause mortality at 90 days. Results: A total of 519 patients (mean age, 69.0 ± 13.4 years; 53.8% males) were included. Patients in the HGHA group had a significantly increased risk of unfavorable functional outcome (OR, 1.81; 95%CI, 1.01–3.23) and mortality (OR, 1.75; 95%CI, 1.01–3.06) at 90 days compared with those in the NGNA group after adjustment for confounders. There was no significant association between NGHA (OR, 0.43; 95%CI, 0.12–1.53) or HGNA (OR, 1.46; 95%CI, 0.84–2.56) and outcomes compared to the NGNA group. Conclusion: The combination of high admission glucose and high HbA1c level was significantly associated with unfavorable functional outcome and mortality at 90 days in AIS patients with reperfusion therapy.

## 1. Introduction

Intravenous thrombolysis (IVT) has been proved to be an effective treatment in improving functional outcomes at 3 months for patients with acute ischemic stroke (AIS) within 4.5 h after onset [1]. Recent studies have demonstrated that endovascular thrombectomy (EVT) with stent retrievers is effective and safe for AIS in patients with large artery occlusion. Some patients who meet indications for both IVT and EVT may undergo bridging therapy [2,3,4,5,6]. AIS patients frequently have elevated glucose levels upon admission [7,8]. Hyperglycemia increases oxidative stress and contributes to blood–brain barrier dysfunction following ischemia reperfusion injury [9]. Previous studies suggested that admission hyperglycemia (aHG) is associated with worse outcomes, including functional dependency, long-term mortality, and hemorrhagic complications, in patients treated with IVT or EVT [10,11,12,13]. However, the above studies did not consider the influence of patients’ pre-stroke chronic hyperglycemia on outcomes.

It has been proved that chronic hyperglycemia is associated with poor functional outcome in patients with acute ischemic stroke [14]. Chronic hyperglycemia is related to small vessel disease and may lead to a worse response to reperfusion therapies. Choi KH et al. reported that chronic hyperglycemia is associated with a significantly higher risk of unfavorable functional outcome at 3 months after EVT in recanalized patients compared with non-recanalized patients [15]. A recent study also demonstrated that increasing HbA1c levels (per 10 mmol/mol) are associated with reduced functional independence [16]. Hemoglobin A1c (HbA1c) is a marker of glycemic control in the past 3 months and a high HbA1c level indicates chronic hyperglycemia before the ischemic stroke [17]. However, it remains unclear whether chronic hyperglycemia influences the association between aHG and worse outcome in patients with reperfusion therapy [10,13].

This study aimed to explore whether HbA1c level and admission glucose level have joint effects on clinical outcomes of AIS patients treated with reperfusion therapy.

## 2. Methods

### 2.1. Study Design and Participants

The study consecutively recruited AIS patients with reperfusion therapy admitted to the Neurology Department of West China Hospital from January 2018 to February 2021. AIS was diagnosed according to the World Health Organization-defined criteria [18]. Patients who met the following inclusion criteria were enrolled: (1) received reperfusion therapy (IVT and/or EVT) and (2) had their glucose level measured on admission and their HbA1c level measured on the second day of hospitalization. Patients were excluded if they were younger than 18 years. This retrospective study was approved by the Scientific Research Department of West China Hospital and patients’ written informed consent was waived since the data were anonymous.

### 2.2. Data Collection

All patients’ information were collected through a review of the medical record on admission, including baseline demographics (age, gender), vascular risk factors (hypertension, diabetes, history of stroke, smoking, alcohol consumption, and atrial fibrillation), preadmission hypoglycemic medications use, methods of reperfusion, National Institute of Health Stroke Scale (NIHSS) score, and symptomatic intracranial hemorrhage (SICH). The measurements of white blood cell count, admission glucose levels, low-density lipoprotein (LDL) levels, and blood pressure were conducted during the first day of hospitalization. Imaging data and diagnostic information were also collected during hospitalization. Non-smoking was defined as never smoked and past smoking [19]. Stress hyperglycemia ratio (SHR) was defined as the admission glucose concentration divided by the estimated average glucose concentration (Estimated mean glucose = 1.59 × HbA1c − 2.59) [20]. Stroke subtypes followed the Trial of Org 10,172 in Acute Stroke Treatment (TOAST) classification [21]. SICH was defined as the presence of a type 2 parenchymal hematoma (PH-2) on brain CT accounting for neurological deterioration according to ECASS III [22]. 

### 2.3. Outcome Assessment

Clinical outcome was assessed by the modified Rankin Scale (mRS) at 3 months by a clinic interview or telephone conversations with the patients or the patient’s relatives. The primary outcome was an unfavorable functional outcome defined as mRS (3–6) at 3 months and the secondary outcome was 3-month mortality [20,23,24].

### 2.4. Statistical Analysis

Categorical variables are expressed as frequencies (%) and were compared in chi-square tests. Continuous variables are described as the mean (SD) or median (interquartile range (Q1–Q3)) and were compared by use of ANOVA or the Wilcoxon rank-sum test. 

Acute hyperglycemia upon admission was defined as a random plasma glucose level > 140 mg/dL (7.8 mmol/L) and high HbA1c levels were defined as plasma HbA1c level > 6.5%, according to a previous study and the current criteria for diagnosing diabetes [12,25]. Participants were divided into four groups by admission glucose level (categorical variable: <7.8 and ≥7.8 mmol/L) and HbA1c level (categorical variable: <6.5% and ≥6.5%): admission glucose < 7.8 and HbA1c < 6.5% (NGNA), admission glucose < 7.8 and HbA1c ≥ 6.5% (NGHA), admission glucose ≥ 7.8 and HbA1c < 6.5% (HGNA), and admission glucose ≥ 7.8 and HbA1c ≥ 6.5% (HGHA). 

Univariable and multivariable logistic regression models were used to investigate the association between admission glucose combined with HbA1c levels and outcomes. We included all potential covariates with *p*-values < 0.10 in the univariable logistic analysis to adjust the multivariable logistic regression models. Patients with NGNH were defined as the reference group. Multivariate ordinal logistic regression was used to estimate the adjusted odds ratio for a shift in the distribution of mRS score between the reference group and other groups.

All tests were two-tailed and a *p*-value of <0.05 was considered statistically significant. Data were analyzed with IBM SPSS Statistical version 25.0 (New York, NY, USA).

## 3. Results

### 3.1. Baseline Characteristics

**Figure 1:**
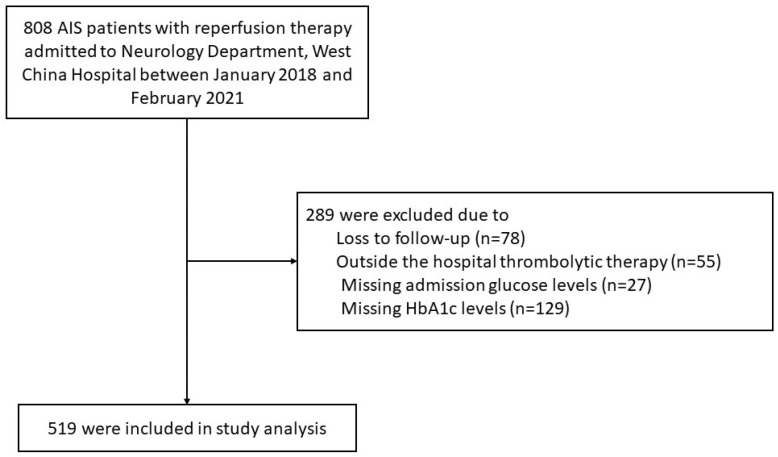
Patients flow chart. AIS, acute ischemic stroke; HbA1c, hemoglobin A1c.

A total of 519 patients were included in the final analysis. A flowchart of patient selection is shown in Figure 1. 

The mean age of the 519 participants was 69 years (SD: 13.4 years); 279 participants (53.8%) were men. The mean level of admission glucose was 8.51 mmol/L (SD: 3.13 mmol/L). A total of 47.4% of patients had aHG. The mean level of HbA1c was 6.39% (SD: 1.35%). A total of 29.9% of patients had high HbA1c levels. Compared with patients in the NGNA group, those in the HGHA group were older, had a higher proportion of males, had higher systolic blood pressure (SBP) and white blood cell (WBC) levels, and had a higher percentage of a history of stroke, hypertension, and diabetes. A total of 79.7% of patients with HbA1c > 6.5 had a history of diabetes. The proportion of Metformin use was higher in the NGHA and HGHA groups than in the NGNA group. The proportion of SICH was the highest in the NGNA group but there was no statistical difference between groups. A total of 294 patients (56.6%) had an unfavorable functional outcome (mRS of 3–6) on 3 months of follow-up. The HGHA group had the highest percentage of death at the 3-month follow-up (Table 1). After Bonferroni correction, SHR was statistically different between any two groups. It was higher in both the HGNA and HGHA groups than in the NGNA group and was highest in the HGNA group (Figure S1 in the Supplementary).

### 3.2. Association between the Combination of Admission Glucose with HbA1c and mRS Score

In the univariable logistic regression analysis, age, gender, baseline NIHSS score, smoking, alcohol consumption, atrial fibrillation, white blood cell count, TOAST classification, and reperfusion therapy method were considered as potential confounders affecting the 3-month unfavorable functional outcome (*p* < 0.10, Table 2). After adjustment for confounders, no significant linear correlation was found between admission glucose or HbA1c and 3-month unfavorable functional outcome (OR, 1.06; 95%CI, 0.99–1.14; *p* = 0.101 and OR, 1.10; 95%CI, 0.93–1.29; *p* = 0.258). The HGHA group had a higher risk of poor outcome than the NGNA group (OR, 1.81; 95%CI, 1.01–3.23; *p* = 0.043). Both admission glucose and HbA1c were independent predictors for 3-month mortality when they were regarded as continuous variables (OR, 1.12; 95%CI, 1.05–1.20; *p* = 0.001 and OR, 1.21; 95%CI, 1.04–1.41; *p* = 0.014). Compared with the NGNA group, a higher risk of 3-month mortality was found only in the HGHA group after adjusting for confounding factors (OR, 1.75; 95%CI, 1.01–3.06; *p* = 0.048, Table 3).

**Figure 2:**
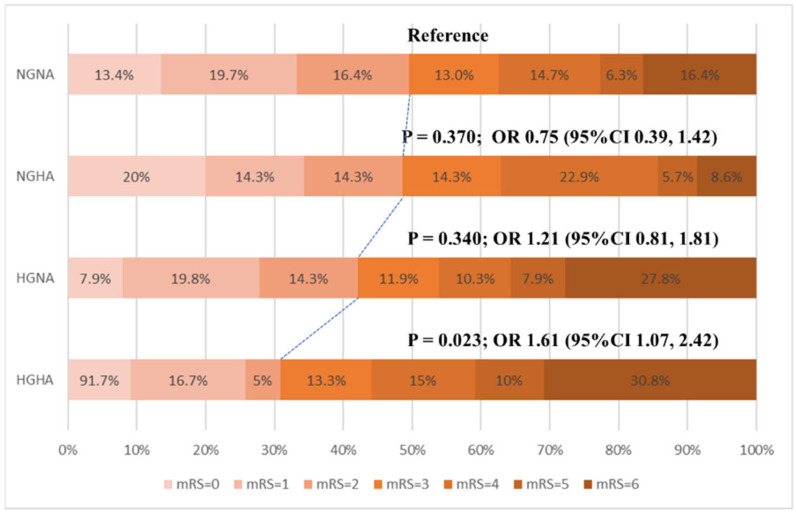
Correlation of different groups and functional improvement (multivariate ordinal logistic regression) according to the distribution of the mRS score at 3 months. Scores range from 0 to 6, with 0 indicating no symptoms, 1 no clinically significant disability, 2 slight disability (patient is able to look after own affairs without assistance but has one or more symptoms), 3 moderate disability (patient requires some help but is able to walk unassisted), 4 moderately severe disability (patient is unable to attend to bodily needs without assistance and is unable to walk unassisted), 5 severe disability (patient requires constant nursing care and attention), and 6 death. NGNA, normal glucose and normal HbA1c; NGHA, normal glucose and high HbA1c; HGNA, high glucose and normal HbA1c; HGHA, normal glucose and high HbA1c; mRS, modified Rankin Scale.

Functional outcome stratified by different glucose and HbA1c levels is shown in Figure 2. There was a shift in the distribution of the mRS in the HGHA group compared with the reference group. After adjusting for age, gender, baseline NIHSS score, smoking, atrial fibrillation, white blood cell count, and reperfusion therapy method, the association between HGHA and mRS score remained significant (OR, 1.61; 95%CI, 1.07–2.42; *p* = 0.023, Figure 2). The shift toward worse outcomes in favor of the intervention was consistent for all categories of the mRS, except for no symptoms (mRS = 0) (Figure 2).

The relationship between SHR and mRS score was also analyzed. After dividing the SHR into four equal quartiles, the other three quartiles were statistically associated with 3-month unfavorable functional outcomes compared with the lowest quartile (OR, 1.95; 95%CI, 1.06–3.57; *p* = 0.037 and OR, 2.19; 95%CI, 1.19–4.04; *p* = 0.014 and OR, 2.14; 95%CI, 1.15–3.99; *p* = 0.022, Table S1). The risk of 3-month unfavorable functional outcome increased stepwise across quartiles (*p* for trend = 0.014). In addition, SHR was positively correlated to 3-month mortality as a continuous variable (OR, 2.21; 95%CI, 1.04–4.69; *p* = 0.040, Table S1).

## 4. Discussion

In this retrospective study involving AIS patients treated with reperfusion therapy, we found that admission glucose levels or HbA1c levels did not have a significant association with 3-month functional outcomes but patients in the HGHA group were associated with an increased risk of unfavorable functional outcomes and all-cause mortality at 3 months.

Previous studies reported that aHG is independently associated with unfavorable functional outcome at 90 days in patients treated with IVT and this result applied to both diabetics and non-diabetics [10,26]. The diabetes group included patients with or without chronic hyperglycemia pre-admission. A high HbA1c level indicates chronic hyperglycemia before the ischemic stroke [10]. Compared to admission glucose, whether HbA1c is independently associated with functional outcome after reperfusion therapy remained controversial in previous studies. In the ACROSS-China registry, authors demonstrated that patients with HbA1c > 6.5 did not show a significant correlation with 1-year poor functional outcome [27]. In some studies, HbA1c was found to be independently associated with worse clinical outcomes in a linear or threshold relationship [12,13]. The joint effect of chronic hyperglycemia rather than diabetes and admission glucose on clinical outcome needs to be further explored. In this study, the HGHA group was found to be associated with an increased risk of unfavorable functional outcomes in AIS patients with reperfusion therapy. Our study did not find a linear relationship between admission glucose level and unfavorable outcomes, which is congruent with a cohort study involving 223 AIS patients with EVT [15]. The results of this study indicate that both admission glucose and HbA1c correlated with 3-month mortality in a linear relationship. Therefore, it can be speculated that, when patients’ admission glucose and HbA1c were both above the threshold, the risk of death increased as the values rose. Earlier studies reported similar results as well [7,13].

The underlying mechanism of this result may be the joint effect of chronic hyperglycemia and acute aHG. High HbA1c levels and high admission glucose levels impact the outcomes in different ways but contribute to the worse functional outcomes. Chronic hyperglycemia causes cerebrovascular injury through mechanisms unrelated to the thrombo-inflammatory changes in acute aHG. It has been demonstrated that diabetes is related to cerebral small vessel disease, which may impair the brain’s ability to compensate for acute ischemic insults [28]. A recent study involving a stroke model in mice with impaired glucose tolerance demonstrated that chronic hyperglycemia is significantly associated with larger infarct volume [29] and this may result in a worse outcome at 3 months [30]. In another study, researchers found that chronic pre-stroke hyperglycemia affected middle cerebral artery blood flow velocity during recovery, which might impact the functional outcomes of patients [31]. Hyperglycemia plays an important role in early reperfusion by increasing blood–brain barrier permeability and extravasation of neutrophils within the infarcted area, leading to worse clinical outcomes [32,33]. Recent studies reported that high blood glucose may be related to a lower cerebral glucose level and contributes to procoagulant platelet formation, which would increase the severity of ischemia [34,35].

In this study, patients in the HGNA group did not have a significantly increased risk of poor outcome. This result is not consistent with previous studies involving the SHR; they demonstrated that a high SHR is related to worse outcomes in AIS patients with or without EVT [14,36,37,38]. A possible explanation for this result might be that patients in the HGHA group had elevated levels of both SHR and HbA1c, which would lead to a worse outcome. 

This study also showed that patients with normal glucose and high HbA1c level were not significantly correlated with unfavorable functional outcome. Patients in this group did not suffer from stress hyperglycemia although they had chronic hyperglycemia pre-admission. According to a previous study, the reason that they did not develop stress hyperglycemia was because the insula of these patients was not damaged [39]. The number of patients in this group is small and the possibility of bias in the results cannot be excluded. Additionally, we found that the NGNA group had a higher proportion of patients with SICH. In this study, the NGNA group had more patients with EVT, which may result in more patients with SICH.

These findings suggest that physicians should control pre-stroke glucose within the normal range in diabetic patients to protect them from the adverse joint effect of HGHA.

Compared with previous studies that focused on a single factor, our study explored which patients would have a worse clinical outcome from another perspective by utilizing different threshold level groupings of admission glucose and HbA1c. The results from our research extend those of previous studies by dividing patients into four groups according to admission glucose and HbA1c, suggesting that the combination of admission glucose and HbA1c is associated with functional outcomes in patients treated with IVT and/or EVT.

There are some potential limitations to our study. First, this was a retrospective study from a single institution and patients whose HbA1c levels were not measured in the first week after admission were excluded, which could have led to selection bias and limit the generalizability of the study findings. Second, dynamic changes in blood glucose and HbA1c at different stages of follow-up were not considered in this study. Third, admission glucose was affected by previous food intake and the time of day and may not reliably reflect acute hyperglycemia [40]. Fourth, we included patients with atherosclerotic and cardiogenic embolic types as well as anterior and posterior circulation occlusion. Patients with cardiogenic embolic types were more likely to have worse outcomes and patients with posterior circulation occlusion were at less risk of EVT, which may have had an impact on the prognosis. Finally, more research is needed to better validate the findings in our study.

## 5. Conclusions

Our study showed that neither admission glucose level nor HbA1c was an independent predictor of unfavorable functional outcomes but the combination of high admission glucose and high HbA1c level was significantly associated with poor clinical outcomes and all-cause mortality at 90 days in AIS patients with reperfusion therapy. This finding lends support to the importance of long-term glycemic control.

## Figures and Tables

**Table 1:** Characteristics of patients according to admission glucose and HbA1c levels.

Characteristics	Overall (*n* = 519)	NGNA (*n* = 238)	NGHA (*n* = 35)	HGNA (*n* = 126)	HGHA (*n* = 120)	*p*-Value
Age, years, mean (SD)	69.0 (13.4)	66.5 (14.5)	71.1 (10.2)	70.0 (13.1)	72.1 (11.1)	0.001
Male, *n* (%)	279 (53.8)	122 (51.3)	24 (68.6)	63 (50.0)	70 (58.3)	0.144
Hypertension, *n* (%)	298 (57.4)	119 (50.0)	22 (62.9)	77 (61.1)	80 (66.7)	0.014
Diabetes, *n* (%)	138 (24.0)	10 (4.2)	21 (60.0)	18 (14.3)	89 (74.2)	<0.001
Atrial fibrillation, *n* (%)	245 (47.2)	104 (43.7)	14 (40.0)	71 (56.3)	56 (46.7)	0.105
History of stroke, *n* (%)	51 (9.8)	15 (6.3)	6 (17.1)	18 (14.3)	12 (10.0)	0.045
Current smoking, *n* (%)	123 (23.7)	55 (23.1)	8 (22.9)	31 (24.6)	29 (24.2)	0.988
Alcohol consumption, *n* (%)	110 (21.2)	48 (20.2)	5 (14.3)	32 (25.4)	25 (20.8)	0.477
Baseline NIHSS, median (Q1–Q3)	13 (8–17)	13 (7–17)	13 (7–16)	14 (9–17)	14 (8-18)	0.194
Systolic pressure, mmHg, mean (SD)	142.7 (26.4)	141.1 (25.9)	145.1 (24.6)	141.4 (27.0)	146.9 (26.9)	0.212
Diastolic pressure, mmHg, mean (SD)	83.0 (17.1)	82.1 (15.7)	83.0 (15.0)	82.6 (18.7)	84.9 (18.5)	0.539
Admission glucose, mmol/L, mean (SD)	8.51 (3.13)	6.47 (0.75)	6.58 (1.10)	9.44 (1.51)	12.15 (3.88)	<0.001
HbA1c, mean (SD)	6.39 (1.35)	5.7 (0.4)	7.3 (1.1)	5.8 (0.4)	8.0 (1.6)	<0.001
SHR, mean (SD)	1.14 (0.29)	1.01 (0.16)	0.75 (0.17)	1.42 (0.25)	1.20 (0.27)	<0.001 ^※^
White Blood Cell *, 10^9^/L, mean (SD)	8.57 (3.38)	7.96 (2.82)	8.75 (3.01)	9.32 (4.07)	8.93 (3.51)	0.002
Low-density lipoprotein, mmol/L, mean (SD)	2.53 (0.92)	2.55 (0.95)	2.46 (0.97)	2.49 (0.89)	2.53 (0.87)	0.896
**TOAST classification, *n* (%)**						0.002
Large-artery atherosclerosis	179 (34.5)	79 (33.2)	16 (45.7)	33 (26.2)	51 (42.5)	
Cardio-embolism	216 (41.6)	97 (40.8)	13 (37.1)	58 (46.0)	48 (40.0)	
Lacunar	31 (6.0)	23 (9.7)	3 (37.1)	3 (2.4)	2 (1.7)	
Other	16 (3.1)	10 (4.2)	0 (0.0)	4 (3.2)	2 (1.7)	
Undetermined	77 (14.8)	29 (12.2)	3 (8.6)	28 (22.2)	17 (14.2)	
**Reperfusion therapy method, *n* (%)**						0.802
Thrombolysis only	165 (31.8)	77(32.4)	13 (37.1)	35 (27.8)	40 (33.3)	
Thrombectomy only	257 (49.5)	121 (50.8)	16 (45.7)	62 (49.2)	58 (48.3)	
Thrombolysis and thrombectomy	97 (18.7)	40 (16.8)	6 (17.1)	29 (23.0)	22 (18.3)	
**Hypoglycemic medication histories**						<0.001
None, *n* (%)	423 (81.5)	235 (98.7)	18 (51.4)	114 (90.5)	56 (46.7)	
Including Metformin, *n* (%)	50 (9.6)	0 (0)	10 (28.6)	6 (4.8)	34 (28.3)	
Other hypoglycemic medications, *n* (%)	46 (8.9)	3 (1.3)	7 (20.0)	6 (4.8)	30 (25.0)	
Symptomatic intracranial hemorrhage, *n* (%)	19 (3.8)	11 (4.7)	0 (0)	3 (2.5)	5 (4.3)	0.284
3-month unfavorable functional outcome (mRS > 2)	294 (56.6)	120 (50.4)	18 (51.4)	73 (57.9)	83 (69.2)	0.008
3-month mortality, *n* (%)	114 (22.0)	39 (16.4)	3 (8.6)	35 (27.8)	37 (30.8)	0.001

NGNA, normal glucose and normal HbA1c; NGHA, normal glucose and high HbA1c; HGNA, high glucose and normal HbA1c; HGHA, normal glucose and high HbA1c; SD, standard deviation; HbA1c, hemoglobin A1c; SHR, stress hyperglycemia ratio (SHR = admission glucose level/[(1.59 × HbA1c) − 2.59]; NIHSS, National Institutes of Health Stroke Scale; TOAST, The Trial of Org 10,172 in Acute Stroke Treatment. * Total white blood cell count in routine blood; ^※^ The Bonferroni correction method was applied to multiple comparisons using a *p*-value < 0.05/number of comparisons as a threshold for statistical significance (*p*-value < 0.008), and *p*-values < 0.001 for all six comparisons.

**Table 2:** Univariable logistic regression analysis of factors associated with 3-month unfavorable outcome.

Variable	Unadjusted Odds Ratio (95% Confidence Internal)	*p*-Value
Age	1.05 (1.03, 1.06)	<0.001
Male	0.49 (0.35, 0.70)	<0.001
Hypertension	1.26 (0.89, 1.79)	0.198
Diabetes	1.27 (0.85, 1.88)	0.243
Atrial fibrillation	2.17 (1.52, 3.10)	<0.001
History of stroke	1.60 (0.87, 2.95)	0.131
Current smoking	0.53 (0.35, 0.80)	0.002
Alcohol consumption	0.65 (0.43, 0.99)	0.044
Baseline NIHSS	1.20 (1.15, 1.24)	<0.001
Admission glucose	1.10 (1.03, 1.17)	0.003
HbA1c	1.12 (0.98, 1.29)	0.094
Systolic pressure	1.00 (1.00, 1.01)	0.238
Diastolic pressure	1.00 (0.99, 1.01)	0.881
White Blood Cell	1.08 (1.03, 1.15)	0.005
Low density lipoprotein	0.88 (0.72, 1.06)	0.177
**TOAST classification**		
Large-artery atherosclerosis	Reference	
Cardio-embolism	1.93 (1.28, 2.90)	0.002
Lacunar	0.13 (0.05, 0.40)	<0.001
Other	1.16 (0.42, 3.26)	0.774
Undetermined	0.98 (0.57, 1.67)	0.934
**Reperfusion therapy method**		
Thrombolysis only	Reference	
Thrombectomy only	2.54 (1.70, 3.79)	<0.001
Thrombolysis and thrombectomy	2.16 (1.30, 3.60)	0.003
**Hypoglycemic medication histories**		
None	Reference	
Including Metformin	0.83 (0.46, 1.50)	0.545
Other hypoglycemic medications	1.31 (0.70, 2.46)	0.396

HbA1c, hemoglobin A1c; NIHSS, National Institutes of Health Stroke Scale; TOAST, The Trial of Org 10,172 in Acute Stroke Treatment.

**Table 3:** Multivariable logistic regression analysis between different groups (divided by admission glucose and HbA1c) and outcomes.

Variable	Unadjusted Model *	Adjusted Model *
3-month unfavorable functional outcome ^†^		
Admission glucose level	1.10 (1.03, 1.17), 0.003	1.06 (0.99, 1.14), 0.101
HbA1c	1.12 (0.98, 1.29), 0.094	1.10 (0.93, 1.29), 0.258
Outcomes group		
NGNA	Reference	Reference
NGHA	1.04 (0.51, 2.12), 0.911	1.00 (0.42, 2.34), 0.990
HGNA	1.35 (0.88, 2.09), 0.172	0.89 (0.52, 1.53), 0.676
HGHA	2.21 (1.39, 3.51), <0.001	1.81 (1.01, 3.23), 0.043
3-month mortality ^‡^		
Admission glucose level	1.15 (1.08, 1.22), <0.001	1.12 (1.05, 1.20), 0.001
HbA1c	1.21 (1.05, 1.39), 0.007	1.21 (1.04, 1.41), 0.014
Outcomes group		
NGNA	Reference	Reference
NGHA	0.48 (1.14, 1.64), 0.241	0.43 (0.12, 1.53), 0.191
HGNA	1.96 (1.17, 3.30), 0.011	1.46 (0.84, 2.56), 0.183
HGHA	2.28 (1.36, 3.82), 0.002	1.75 (1.01, 3.06), 0.048

* Results for each model are presented as the odds ratio (95% confidence interval), *p*-value. ^†^ Adjusted model: adjusted for age, gender, atrial fibrillation, current smoking, alcohol consumption, baseline NIHSS score, white blood cell, TOAST classification, and reperfusion therapy method. ^‡^ Adjusted model: adjusted for age, gender, baseline NIHSS score, white blood cell, and TOAST classification. NGNA, normal glucose and normal HbA1c; NGHA, normal glucose and high HbA1c; HGNA, high glucose and normal HbA1c; HGHA, high glucose and high HbA1c; NIHSS, National Institutes of Health Stroke Scale; TOAST, The Trial of Org 10,172 in Acute Stroke Treatment.

## Data Availability

The data that support the findings of this study are available from the corresponding author upon reasonable request.

## Supplementary Materials

The following supporting information can be downloaded at: https://www.mdpi.com/article/10.3390/brainsci12020294/s1: Figure S1: Mean SHR in the four groups. * *p*-value < 0.05 after Bonferroni adjustment. SHR, stress hyperglycemic ratio; NGNA, normal glucose and normal HbA1c; NGHA, normal glucose and high HbA1c; HGNA, high glucose and normal HbA1c; HGHA, normal glucose and high HbA1c. CI, confidence interval; Table S1: Multivariable logistic regression analysis between subcategorized SHR groups and outcomes *.

## References

[1] National Institute of Neurological Disorders and Stroke rt-PA Stroke Study Group (1995). Tissue plasminogen activator for acute ischemic stroke. N. Engl. J. Med..

[2] Berkhemer O.A., Fransen P.S., Beumer D., van den Berg L.A., Lingsma H.F., Yoo A.J., Schonewille W.J., Vos J.A., Nederkoorn P.J., Wermer M.J. (2015). A randomized trial of intraarterial treatment for acute ischemic stroke. N. Engl. J. Med..

[3] Campbell B.C., Mitchell P.J., Kleinig T.J., Dewey H.M., Churilov L., Yassi N., Yan B., Dowling R.J., Parsons M.W., Oxley T.J. (2015). Endovascular therapy for ischemic stroke with perfusion-imaging selection. N. Engl. J. Med..

[4] Goyal M., Demchuk A.M., Menon B.K., Eesa M., Rempel J.L., Thornton J., Roy D., Jovin T.G., Willinsky R.A., Sapkota B.L. (2015). Randomized assessment of rapid endovascular treatment of ischemic stroke. N. Engl. J. Med..

[5] Jovin T.G., Chamorro A., Cobo E., de Miquel M.A., Molina C.A., Rovira A., San Román L., Serena J., Abilleira S., Ribó M. (2015). Thrombectomy within 8 hours after symptom onset in ischemic stroke. N. Engl. J. Med..

[6] Saver J.L., Goyal M., Bonafe A., Diener H.C., Levy E.I., Pereira V.M., Albers G.W., Cognard C., Cohen D.J., Hacke W. (2015). Stent-retriever thrombectomy after intravenous t-PA vs. t-PA alone in stroke. N. Engl. J. Med..

[7] Williams L.S., Rotich J., Qi R., Fineberg N., Espay A., Bruno A., Fineberg S.E., Tierney W.R. (2002). Effects of admission hyperglycemia on mortality and costs in acute ischemic stroke. Neurology.

[8] Kruyt N.D., Biessels G.J., Devries J.H., Roos Y.B. (2010). Hyperglycemia in acute ischemic stroke: Pathophysiology and clinical management. Nat. Rev. Neurol..

[9] Kamada H., Yu F., Nito C., Chan P.H. (2007). Influence of hyperglycemia on oxidative stress and matrix metalloproteinase-9 activation after focal cerebral ischemia/reperfusion in rats: Relation to blood-brain barrier dysfunction. Stroke.

[10] Tsivgoulis G., Katsanos A.H., Mavridis D., Lambadiari V., Roffe C., Macleod M.J., Sevcik P., Cappellari M., Nevšímalová M., Toni D. (2019). Association of Baseline Hyperglycemia with Outcomes of Patients with and without Diabetes with Acute Ischemic Stroke Treated with Intravenous Thrombolysis: A Propensity Score-Matched Analysis from the SITS-ISTR Registry. Diabetes.

[11] Lin S.F., Chao A.C., Hu H.H., Lin R.T., Chen C.H., Chan L., Lin H.J., Sun Y., Lin Y.Y., Chen P.L. (2018). Hyperglycemia predicts unfavorable outcomes in acute ischemic stroke patients treated with intravenous thrombolysis among a Chinese population: A prospective cohort study. J. Neurol. Sci..

[12] Rinkel L.A., Nguyen T.T.M., Guglielmi V., Groot A.E., Posthuma L., Roos Y.B.W.E.M., Majoie C.B.L.M., Lycklama À Nijeholt G.J., Emmer B.J., Multicenter Randomized Clinical Trial of Endovascular Treatment of Acute Ischemic Stroke in the Netherlands (MR CLEAN) Registry Investigators (2020). High Admission Glucose Is Associated with Poor Outcome after Endovascular Treatment for Ischemic Stroke. Stroke.

[13] Luitse M.J., Velthuis B.K., Kappelle L.J., van der Graaf Y., Biessels G.J., DUST Study Group (2017). Chronic hyperglycemia is related to poor functional outcome after acute ischemic stroke. Int. J. Stroke.

[14] Goyal N., Tsivgoulis G., Pandhi A., Dillard K., Katsanos A.H., Magoufis G., Chang J.J., Zand R., Hoit D., Safouris A. (2018). Admission hyperglycemia and outcomes in large vessel occlusion strokes treated with mechanical thrombectomy. J. Neurointerv. Surg..

[15] Choi K.H., Kim J.H., Kang K.W., Kim J.T., Choi S.M., Lee S.H., Park M.S., Kim B.C., Kim M.K., Cho K.H. (2019). HbA1c (Glycated Hemoglobin) Levels and Clinical Outcome Post-Mechanical Thrombectomy in Patients with Large Vessel Occlusion. Stroke.

[16] Diprose W.K., Wang M.T.M., McFetridge A., Sutcliffe J., Barber P.A. (2020). Glycated hemoglobin (HbA1c) and outcome following endovascular thrombectomy for ischemic stroke. J. Neurointerv. Surg..

[17] Goldstein D.E., Little R.R., Lorenz R.A., Malone J.I., Nathan D., Peterson C.M., Sacks D.B. (2004). Tests of glycemia in diabetes. Diabetes Care.

[18] WHO MONICA Project Principal Investigators (1988). The World Health Organization MONICA Project (monitoring trends and determinants in cardiovascular disease): A major international collaboration. J. Clin. Epidemiol..

[19] Lou P., Chen P., Zhang L., Zhang P., Yu J., Zhang N., Wu H., Zhao J. (2012). Relation of sleep quality and sleep duration to type 2 diabetes: A population-based cross-sectional survey. BMJ Open.

[20] Chen X., Liu Z., Miao J., Zheng W., Yang Q., Ye X., Zhuang X., Peng F. (2019). High Stress Hyperglycemia Ratio Predicts Poor Outcome after Mechanical Thrombectomy for Ischemic Stroke. J. Stroke Cerebrovasc. Dis..

[21] Adams H.P., Bendixen B.H., Kappelle L.J., Biller J., Love B.B., Gordon D.L., Marsh E.E. (1993). Classification of subtype of acute ischemic stroke. Definitions for use in a multicenter clinical trial. TOAST. Trial of Org 10172 in Acute Stroke Treatment. Stroke.

[22] Hacke W., Kaste M., Bluhmki E., Brozman M., Dávalos A., Guidetti D., Larrue V., Lees K.R., Medeghri Z., Machnig T. (2008). Thrombolysis with alteplase 3 to 4.5 hours after acute ischemic stroke. N. Engl. J. Med..

[23] Boisseau W., Desilles J.P., Fahed R., Kyheng M., Zuber K., Sabben C., Taylor G., Ben Maacha M., Maier B., Botta D. (2019). Neutrophil count predicts poor outcome despite recanalization after endovascular therapy. Neurology.

[24] Osei E., den Hertog H.M., Berkhemer O.A., Fransen P.S.S., Roos Y.B.W.E.M., Beumer D., van Oostenbrugge R.J., Schonewille W.J., Boiten J., Zandbergen A.A.M. (2017). Admission Glucose and Effect of Intra-Arterial Treatment in Patients with Acute Ischemic Stroke. Stroke.

[25] American Diabetes Association (2020). 2. Classification and Diagnosis of Diabetes: Standards of Medical Care in Diabetes-2020. Diabetes Care.

[26] Poppe A.Y., Majumdar S.R., Jeerakathil T., Ghali W., Buchan A.M., Hill M.D., Canadian Alteplase for Stroke Effectiveness Study Investigators (2009). Admission hyperglycemia predicts a worse outcome in stroke patients treated with intravenous thrombolysis. Diabetes Care.

[27] Jing J., Pan Y., Zhao X., Zheng H., Jia Q., Li H., Guan L., Liu L., Wang C., Meng X. (2016). Prognosis of Ischemic Stroke with Newly Diagnosed Diabetes Mellitus According to Hemoglobin A1c Criteria in Chinese Population. Stroke.

[28] Shukla V., Shakya A.K., Perez-Pinzon M.A., Dave K.R. (2017). Cerebral ischemic damage in diabetes: An inflammatory perspective. J. Neuroinflamm..

[29] Grisotto C., Taïlé J., Planesse C., Diotel N., Gonthier M.P., Meilhac O., Couret D. (2021). High-Fat Diet Aggravates Cerebral Infarct, Hemorrhagic Transformation and Neuroinflammation in a Mouse Stroke Model. Int. J. Mol. Sci..

[30] Ospel J.M., Hill M.D., Menon B.K., Demchuk A., McTaggart R., Nogueira R., Poppe A., Haussen D., Qiu W., Mayank A. (2021). Strength of Association between Infarct Volume and Clinical Outcome Depends on the Magnitude of Infarct Size: Results from the ESCAPE-NA1 Trial. AJNR Am. J. Neuroradiol..

[31] Kaufman C.S., Bai S.X., Eickmeyer S.M., Billinger S.A. (2021). Chronic hyperglycemia before acute ischemic stroke impairs the bilateral cerebrovascular response to exercise during the subacute recovery period. Brain Behav..

[32] Zhang S., An Q., Wang T., Gao S., Zhou G. (2018). Autophagy- and MMP-2/9-mediated Reduction and Redistribution of ZO-1 Contribute to Hyperglycemia-increased Blood-Brain Barrier Permeability During Early Reperfusion in Stroke. Neuroscience.

[33] Couret D., Bourane S., Catan A., Nativel B., Planesse C., Dorsemans A.C., Ait-Arsa I., Cournot M., Rondeau P., Patche J. (2018). A hemorrhagic transformation model of mechanical stroke therapy with acute hyperglycemia in mice. J. Comp. Neurol..

[34] Zhang S., Zuo W., Guo X.F., He W.B., Chen N.H. (2014). Cerebral glucose transporter: The possible therapeutic target for ischemic stroke. Neurochem. Int..

[35] Denorme F., Portier I., Kosaka Y., Campbell R.A. (2021). Hyperglycemia exacerbates ischemic stroke outcome independent of platelet glucose uptake. J. Thromb. Haemost..

[36] Roberts G.W., Quinn S.J., Valentine N., Alhawassi T., O’Dea H., Stranks S.N., Burt M.G., Doogue M.P. (2015). Relative Hyperglycemia, a Marker of Critical Illness: Introducing the Stress Hyperglycemia Ratio. J. Clin. Endocrinol. Metab..

[37] Yuan C., Chen S., Ruan Y., Liu Y., Cheng H., Zeng Y., Chen Y., Cheng Q., Huang G., He W. (2021). The Stress Hyperglycemia Ratio is Associated with Hemorrhagic Transformation in Patients with Acute Ischemic Stroke. Clin. Interv. Aging.

[38] Li J., Quan K., Wang Y., Zhao X., Li Z., Pan Y., Li H., Liu L., Wang Y. (2020). Effect of Stress Hyperglycemia on Neurological Deficit and Mortality in the Acute Ischemic Stroke People with and without Diabetes. Front. Neurol..

[39] Allport L.E., Butcher K.S., Baird T.A., MacGregor L., Desmond P.M., Tress B.M., Colman P., Davis S.M. (2004). Insular cortical ischemia is independently associated with acute stress hyperglycemia. Stroke.

[40] Wnuk M., Popiela T., Drabik L., Brzegowy P., Lasocha B., Wloch-Kopec D., Pulyk R., Jagiella J., Wiacek M., Kaczorowski R. (2020). Fasting Hyperglycemia and Long-term Outcome in Patients with Acute Ischemic Stroke Treated with Mechanical Thrombectomy. J. Stroke Cerebrovasc. Dis..

