# Supplementary material for: Prognostic Significance of Admission Glucose Combined with Hemoglobin A1c in Acute Ischemic Stroke Patients with Reperfusion Therapy
Source: Brain Sci. 2022 Feb 21;12(2):294. doi: 10.3390/brainsci12020294 (PMC8869904; doi:10.3390/brainsci12020294)
Supplement: Supplementary file 1 [file brainsci-12-00294-s001.zip › brainsci-1578813-supplementary.pdf]

Supplementary Materials

Table S1. Multivariable logistic regression analysis between SHR subcategorized groups and outcomes *.

Variable	Unadjusted model	Adjusted model
3-month unfavorable functional outcome†		
SHR	2.52 (1.34, 4.72), 0.004	2.03 (0.93, 4.40), 0.074
Q1 (0-0.94)	Reference	Reference
Q2 (0.94-1.09)	1.75 (1.08, 2.84), 0.024	1.95 (1.06, 3.57), 0.037
Q3 (1.09-1.30)	2.22 (1.35, 3.66), 0.002	2.19 (1.19, 4.04), 0.014
Q4 (1.30-2.27)	2.34 (1.42, 3.84), <0.001	2.14 (1.15, 3.99), 0.022
p for trend	<0.001	0.014
3-month mortality‡		
SHR	2.52 (1.34, 4.72), 0.004	2.21 (1.04, 4.69), 0.040
Q1 (0-0.94)	Reference	Reference
Q2 (0.94-1.09)	1.67 (0.86, 3.25), 0.130	1.62 (0.80, 3.26), 0.178
Q3 (1.09-1.30)	2.50 (1.31, 4.76), 0.006	2.13 (1.08, 4.19), 0.028
Q4 (1.30-2.27)	2.91 (1.54, 5.48), <0.001	2.23 (1.14, 4.40), 0.020
p for trend	<0.001	0.015

* Results for each model are presented as odds ratio (95% confidence interval), p-value. †Adjusted model: adjusted for age, gender, atrial fibrillation, current smoking, alcohol consumption, baseline NIHSS score, white blood cell, TOAST classification and reperfusion therapy method. ‡Adjusted model: adjusted for age, gender, baseline NIHSS score, white blood cell, TOAST classification. SHR, stress hyperglycemic ratio.

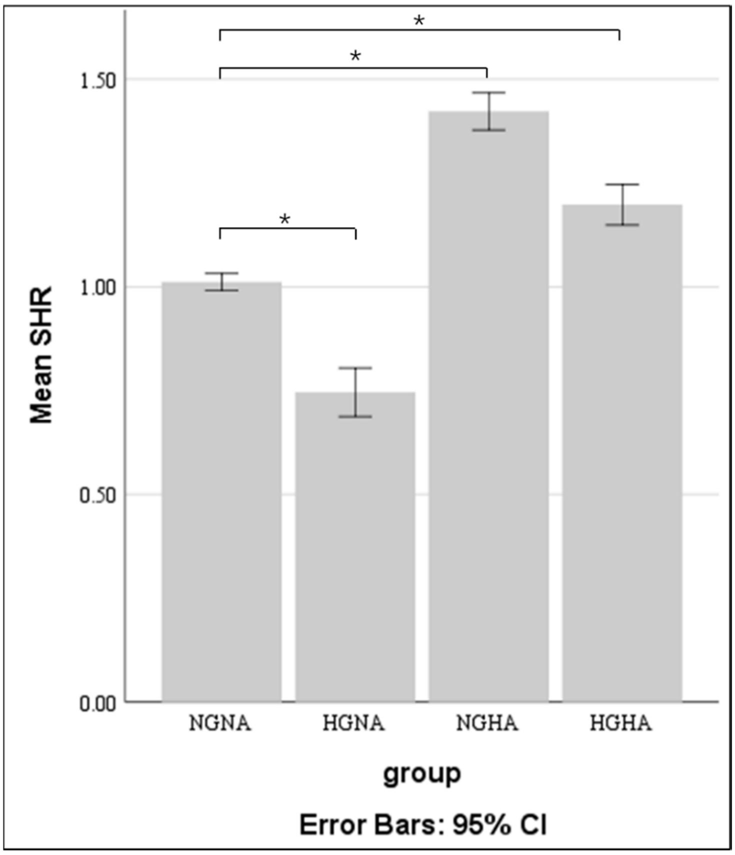

Figure S1. Mean SHR in 4 groups. *p-value < 0.05 after Bonferroni adjustment. SHR, stress hyperglycemic ratio; NGNA, normal glucose and normal HbA1c; NGHA, normal glucose and high HbA1c; HGNA, high glucose and normal HbA1c; HGHA, normal glucose and high HbA1c. CI, confidence interval.
